# Myocardial mechano-energetic efficiency and insulin resistance in non-diabetic members of the Strong Heart Study cohort

**DOI:** 10.1186/s12933-019-0862-9

**Published:** 2019-04-30

**Authors:** Costantino Mancusi, Giovanni de Simone, Lyle G. Best, Wenyu Wang, Ying Zhang, Mary J. Roman, Elisa T. Lee, Barbara V. Howard, Richard B. Devereux

**Affiliations:** 1 Hypertension Research Center & Department of Advanced Biomedical Sciences, Federico II University Hospital, via S.Pansini 5, bld #2, 80131 Naples, Italy; 2 Weill Cornell Med, New York, NY USA; 3 Epidemiology Department, Missouri Breaks Industries Research Inc, Timber Lake, SD USA; 4 Univ of Oklahoma Health Sciences Ctr, Oklahoma City, OK USA; 5 Georgetown-Howard Universities Ctr for Clinical and Translational Science, Washington, DC USA

**Keywords:** Cardiac function, Stroke volume, Myocardial metabolism, Echocardiography

## Abstract

**Background:**

Myocardial energetic efficiency (MEE), is a strong predictor of CV events in hypertensive patient and is reduced in patients with diabetes and metabolic syndrome. We hypothesized that severity of insulin resistance (by HOMA-IR) negatively influences MEE in participants from the Strong Heart Study (SHS).

**Methods:**

We selected non-diabetic participants (n = 3128, 47 ± 17 years, 1807 women, 1447 obese, 870 hypertensive) free of cardiovascular (CV) disease, by merging two cohorts (Strong Heart Study and Strong Heart Family Study, age range 18–93). MEE was estimated as stroke work (SW = systolic blood pressure [SBP] × stroke volume [SV])/“double product” of SBP × heart rate (HR), as an estimate of O_2_ consumption, which can be simplified as SV/HR ratio and expressed in ml/sec. Due to the strong correlation, MEE was normalized by left ventricular (LV) mass (MEEi).

**Results:**

Linear trend analyses showed that with increasing quartiles of HOMA-IR patients were older, more likely to be women, obese and hypertensive, with a trend toward a worse lipid profile (all p for trend < 0.001), progressive increase in LV mass index, stroke index and cardiac index and decline of wall mechanics (all p < 0.0001). In multivariable regression, after adjusting for confounders, and including a kinship coefficient to correct for relatedness, MEEi was negatively associated with HOMA-IR, independently of significant associations with age, sex, blood pressure, lipid profile and central obesity (all p < 0.0001).

**Conclusions:**

Severity of insulin resistance has significant and independent negative impact on myocardial mechano-energetic efficiency in nondiabetic individual from a population study of American Indians.

*Trial registration number* NCT00005134, Name of registry: Strong Heart Study, URL of registry: https://clinicaltrials.gov/ct2/show/NCT00005134, Date of registration: May 25, 2000, Date of enrolment of the first participant to the trial: September 1988

**Electronic supplementary material:**

The online version of this article (10.1186/s12933-019-0862-9) contains supplementary material, which is available to authorized users.

## Background

Left ventricular (LV) work can be represented dimensionally by the force needed to eject blood (stroke volume, SV) into the aorta, and estimated as stroke work (SW), the product of peak-systolic pressure times SV. Cuff systolic blood pressure (SBP) may be used as a surrogate of peak-systolic pressure, under the assumption that the kinetic energy is negligible at least in resting conditions. Thus, at rest, SW may be effectively estimated using fully non-invasive methods.

Energy to support cardiac work is provided almost exclusively by aerobic oxidation of substrate, with close coupling between myocardial oxygen consumption (MVO_2_) and LV structure and function [1]. Thus, the efficiency of the left ventricle in pumping blood into the arterial tree (LV pump performance) may be defined as the ratio between the developed external work (i.e. SW) and the amount of energy produced for each contraction [2].

The energy produced by cardiomyocytes is not entirely converted into external power. Under normal conditions, the proportion of produced energy that is used for contraction is approximately 25%, and the residual energy is mainly dissipated as heat [3]. The ratio between external work delivered by cardiomyocytes and the amount of total energy produced at each beat is, therefore, a measure of myocardial mechano-energetic efficiency (MEE).

We developed a simple method for non-invasive, ultrasound-guided estimation of myocardial mechano-energetic efficiency per gram of LV mass (MEEi), which has been prognostically validated [4, 5]. Low levels of MEEi predict increased incidence of composite cardiovascular events in a large hypertensive population from an open registry in the Campania district in Southern Italy [5] and are associated with high prevalence of obesity and diabetes. At this time, however, there is no information on whether increasing insulin-resistance is a factor compromising MEEi, which can at least in part explain the association with CV morbidity [6].

Accordingly, in this analysis, we tested the hypothesis that MEEi progressively deteriorates for increasing degrees of insulin resistance.

## Methods

### Population sample

We selected non diabetic participants (i.e. no history of diabetes and plasma glucose < 126 mg/dl) from the Strong Heart Study (SHS) initial cohort (2nd exam) and the Strong Heart Family Study (SHFS) cohort (4th exam, age range 18–93), with available data on fasting glucose and fasting insulin levels, and free of prevalent CV disease, as already done in a previous study [7]. Detailed descriptions of the study design and methods of the SHS and SHFS have previously been reported [8–10]. Obesity was classified as BMI ≥ 30 kg/m^2^. Arterial hypertension was defined by BP ≥ 140/90 mmHg or current antihypertensive treatment.

### Measurements

Fasting plasma glucose, lipid profile and other laboratory variables were measured by standard methods, as previously reported [8, 9, 11]. Degree of insulin resistance was assessed using HOMA-IR [12]. Glomerular filtration rate (GFR) was estimated by the simplified Modification of Diet in Renal Disease formula [13].

Echocardiograms were performed using phased-array, commercially available echocardiographs, with M-mode, two-dimensional and Doppler capabilities, and read off line using working stations equipped with frame-grabber to measure on stop-frame images, as previously reported in detail [10]. LV mass, and LV mass index (by normalization for height in m^2.7^) were estimated [10, 14]. Relative wall thickness was computed as a dimensionless ratio between posterior wall thickness and LV internal radius, as the measure of LV concentricity [15]. Stroke volume (SV) was calculated as the difference between LV end-diastolic and end-systolic volumes by the z-derived method, and allometrically normalized by height [16]. Cardiac output was calculated by SV times heart rate and allometrically normalized by height [16]. Ejection fraction and midwall shortening were calculated as previously reported [17]. To estimate MEE, MVO_2_ was approximated using the “double product” of heart rate (HR) times SBP. SW was estimated as SBP × SV. Accordingly, MEE is the ratio between the SW and MVO_2_. Thus:

$${\text{MEE}} = \frac{{\text{SW}}}{{{{\text{MVO}}}_{2} }} = \frac{{{{\text{SBP}}} \times {{\text{SV}}}}}{{{{\text{SBP}}} \times {{\text{HR}}}}} = \frac{{\text{SV}}}{{\text{HR}}}$$

were HR can be expressed in seconds, as the time of one cardiac cycle (HR/60). Thus, MEE could be measured as the ideal amount of blood pumped by one single heart beat in 1 s. However, as we have previously shown, this amount is strictly related to the amount of myocardium available for pump performance [4]. Thus, ratiometric normalization of MEE for LV mass (MEEi) provides the estimate of the ideal amount of blood pumped by each gram of LV mass in 1 s [4, 5].

### Statistics

The population sample was divided into quartiles of HOMA-IR and exploratory statistics were performed to analyse the linear trend among the different degrees of insulin resistance for age, sex, heart rate, blood pressure, BMI, risk profile (including obesity, lipid profile, kidney function), and LV structural and functional parameters (LV mass index and relative wall thickness, stroke index, cardiac index and ejection fraction). ANCOVA was used to study the correlation of MEEi with HOMA-IR, adjusting for age, sex, obesity and hypertension. Because in this population, including members of the SHFS cohort, the level of family relatedness could be significant [18], we also adjusted analysis for a standard kinship coefficient, based on the level of relatedness within family, as previously reported [19]. Continuous variables were used to model independent correlates of MEEi, including HOMA-IR, kinship coefficient, age, sex, systolic BP, plasma cholesterol, triglycerides, waist circumference and two markers of inflammation, fibrinogen and PAI-1. A two-tailed p value < 0.05 was taken as statistically significant.

## Results

The study population comprised 3128 non-diabetic participants (age 47 ± 17 years, 1807 women, 1447 obese, 870 hypertensives). Table 1 shows that with increasing HOMA-IR patients were older, more likely to be women, obese and hypertensive (all p for trend < 0.001). There was also a clear trend toward a progressive increase in blood pressure and heart rate and worse lipid profile with increasing HOMA-IR (all p for trend < 0.001).

**Table 1:** Demographics and metabolic risk profile in quartiles of HOMA-IR

	≤ 1.71 (n = 784)	1.72–2.75 (n = 776)	2.76–4.66 (n = 785)	≥ 4.67 (n = 783)
Age (years)*	44 ± 18	46 ± 18	48 ± 16	48 ± 16
Sex (% women)^†^	52	58	59	62
Hypertension (%)^†^	18	28	31	35
Obesity (%)^†^	12	37	58	78
Body mass index (kg/m^2^)^†^	25 ± 4	29 ± 5	31 ± 5	35 ± 7
Waist circumference (cm)^†^	88 ± 12	99 ± 12	103 ± 13	113 ± 15
Systolic BP (mmHg)^†^	119 ± 18	122 ± 17	124 ± 16	126 ± 17
Diastolic BP (mmHg)^†^	72 ± 11	75 ± 11	76 ± 10	77 ± 10
Heart rate (bpm)^†^	67 ± 11	68 ± 11	69 ± 11	70 ± 11
GFR_MDRD_ (ml/min/1.73 m^2^)*	94 ± 27	92 ± 25	92 ± 47	93 ± 27
Cholesterol (mg/dl)*	181 ± 38	190 ± 38	192 ± 37	185 ± 36
HDL-c (mg/dl)^†^	55 ± 17	50 ± 15	46 ± 13	42 ± 12
Triglycerides (mg/dl)^†^	105 ± 55	139 ± 87	161 ± 92	166 ± 105
Fibrinogen (mg/dl)^†^	335 ± 70	348 ± 74	360 ± 76	367 ± 77
PAI-1 (ng/ml)^†^	40 ± 47	48 ± 36	57 ± 40	71 ± 47

*p for linear trend < 0.01

^†^p for trend < 0.001

**Fig. 1:**
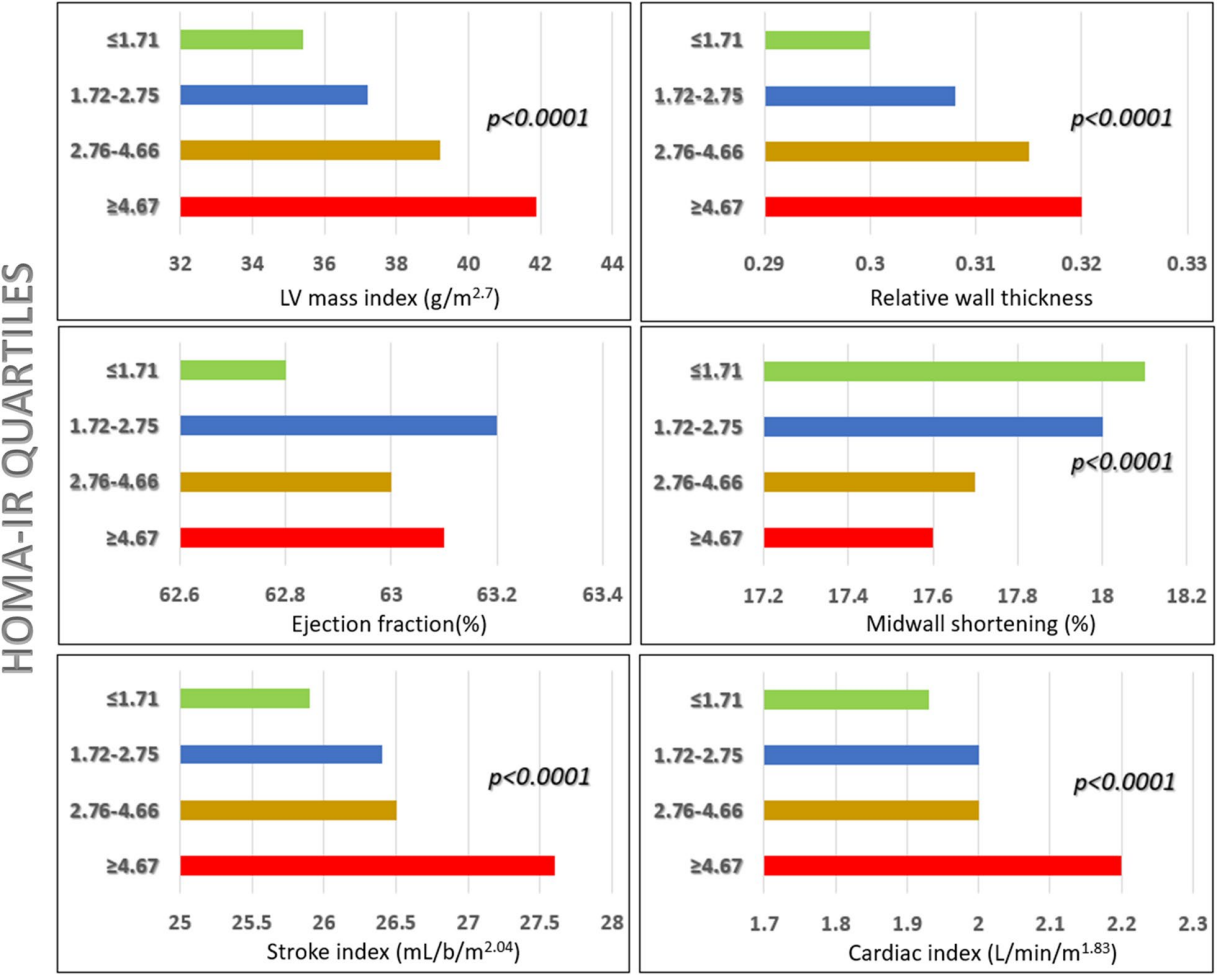
LV geometry, systolic function and performance in quartiles of HOMA-IR

**Fig. 2:**
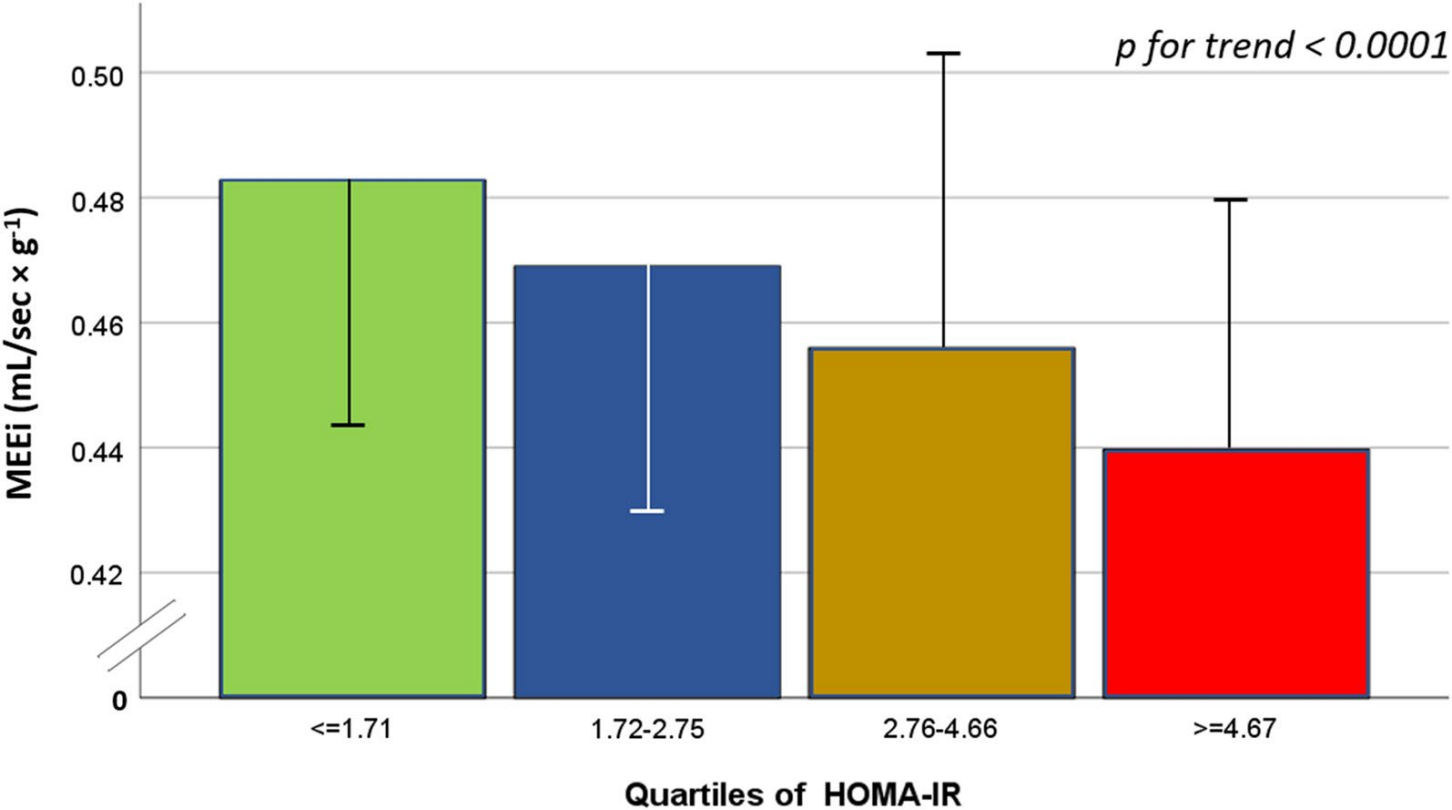
Least square means of MEEi for quartiles of HOMA-IR (insulin resistance), after adjusting for family relatedness, age, sex, obesity and hypertension. MEEi progressively decreases with increasing HOMA-IR

Whereas no effect was observed in ejection fraction, increasing in HOMA-IR was associated with progressive increase in LV mass index, stroke index and cardiac index and decline of midwall shortening (all p < 0.0001) (Fig. 1 and Additional file 1: Table S1). After adjusting for the kinship coefficient, age, sex, obesity and hypertension, MEEi progressively decreased with increasing HOMA-IR (Fig. 2).

In sequential multivariable regression models, the correlation of MEEi with HOMA-IR was adjusted for many potential covariates (Table 2). First, we run the model including family relatedness. This regression model demonstrated that the negative relation between MEEi and HOMA-IR was independent of the significant effect of kinship coefficient. In the additional models by adding sequentially demographics, risk factors and markers of inflammation, HOMA-IR remained highly significant (all p < 0.0001).

**Table 2:** Models of multiple linear regression between HOMA-IR and MEEi, adjusting for kinship coefficient and subsequently for age and sex, risk factors and finally markers of inflammation

	Adjusted for kinship coefficient		+Adjusted for age and sex		+Adjusted for CV risk factors		+Adjusted for markers of inflammation	
	Standardized β-coefficients	p≤	Standardized β-coefficients	p≤	Standardized β-coefficients	p≤	Standardized β-coefficients	p≤
Kinship coefficient	0.295	0.0001	0.069	0.003	0.079	0.0001	0.087	0.0001
HOMA-IR	− 0.166	0.0001	− 0.176	0.0001	− 0.078	0.0001	− 0.070	0.0001
Age (years)			− 0.342	0.0001	− 0.236	0.0001	− 0.239	0.0001
Sex (M/F)			0.155	0.0001	0.130	0.0001	0.144	0.0001
Systolic BP (mmHg)					− 0.151	0.0001	− 0.151	0.0001
Cholesterol (mg/dl)					− 0.037	0.055	− 0.039	0.042
Triglycerides (mg/dl)					− 0.042	0.025	− 0.034	0.069
Waist circumference (cm)					− 0.196	0.0001	− 0.156	0.0001
GFR_MDRD_ (ml/min/1.73 m^2^)					0.013	0.462	0.016	0.357
Fibrinogen (mg/dl)							− 0.077	0.0001
PAI-1 (ng/ml)							− 0.075	0.0001

*BP* blood pressure

## Discussion

This study demonstrates that in non-diabetic participants of the SHS cohort with normal ejection fraction and free of prevalent CV disease, insulin resistance is a significant contributor of the variance of myocardial mechano-energetic efficiency per gram of LV mass. The effect of insulin resistance could be demonstrated to be independent of major CV risk factors, including hypertension, lipid profile and central obesity, all factors linked to metabolic syndrome that could mediate the direct relation between insulin resistance and myocardial energetic efficiency [20]. This is a novel finding, consistent with the evidence that MEEi is emerging as a potent marker of CV risk [5].

In a normal myocardium, 60–70% of energy is produced by fatty acid oxidation, while only 30–40% of energy is produced by glucose-pyruvate oxidation. There are major differences between the two pathways [21]. One molecule of fatty acid produces 105 molecules of ATP using 46 atoms of O_2_. In contrast, one molecule of glucose produces only 31 ATPs but using only 12 atoms of O_2_. Thus, the ratio of produced ATP/MVO_2_ is higher with glucose (P/O = 2.58) than with fatty acids (P/O = 2.28), which produces a redundant number of ATP molecules, a source of energy that is mainly dissipated as heat [22, 23].

Accordingly, myocardial mechano-energetic efficiency is much higher when utilizing glucose than with fatty acids. In the diseased heart, there is in fact a progressive shift toward more glucose utilization up to a near total utilization in heart failure, to realize the most efficient energetic mechanisms in conditions of distress [23]. According to the described scenario, the consequence of insulin resistance at the myocardial level is the inconvenient enhancement of fatty acid oxidation to maintain energy production. Experiments in rats [24–26] and evidence in humans [27] confirm this shift toward fatty acid metabolism. This shift is deleterious especially in pathological conditions, when the natural energy provider should be glucose/lactate oxidation [23, 28]. In addition, increased fatty acid utilization promotes synthesis of proteins that alter mitochondrial mechanism, resulting in more energy dissipated as heat [29].

Taken together, our findings support the assumption that mechanisms of production and delivery of energy play substantial role in the evolution of at least some clinical manifestation of overt CV disease.

**Fig. 3:**
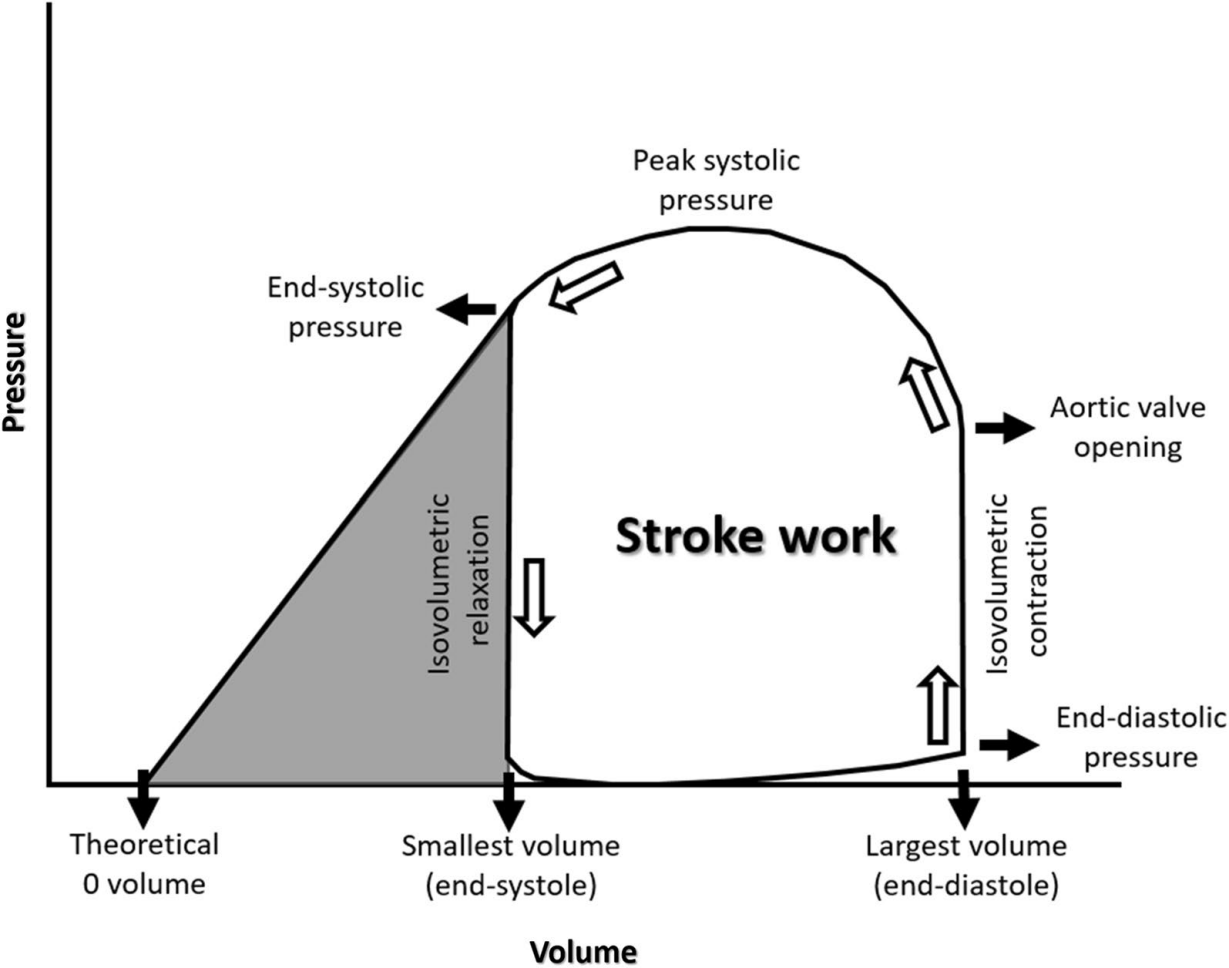
Pressure–volume loop with the indication of the different steps of cardiac cycle, starting with end-systolic volume and the lowest cavity pressure. Cardiac activity proceeds counterclockwise. Stroke work is the area of the loop than can be approximated as a dimensionless rectangle area with the basis represented by stroke volume and the height by peak-systolic pressure. The grey area is the potential energy developed

Compared to our indirect approach, a direct measure of myocardial metabolism would be desirable. However, direct detection of myocardial energetic metabolism is not feasible on epidemiological scale. The approach we use to estimate myocardial mechano-energetic efficiency has a strong rationale. Systolic work at each cardiac beat (stroke work) is represented as the area of the pressure–volume loop (Fig. 3). As can be seen in the figure, this area can be approximated by a dimensionless rectangle having for base the volume variation (i.e. stroke volume) and for height the peak-systolic pressure. This calculation has been done, invasively validated and largely adopted [30], and should be considered well representative of LV external systolic work. The pressure volume loop also helps explaining the efficacy of the double product to estimate MVO_2_ [31, 32]. A substantial part of MVO_2_ is devoted to the development of isovolumic tension that alters geometry of elastic elements before ejection. The isovolumic activity, therefore, do not develop any real physical work, while dissipating energy. This part of energy waste increases with increasing heart rate, mainly due to the increased frequency of isovolumic contraction, while it is reduced if cardiac output is sustained by stroke volume. With the energetic sparing obtained by increasing stroke volume, if aortic pressure increases, end-systolic wall tension also increases, and energy dissipation also increases. For these reasons, product of heart rate by systolic pressure is a potent and reliable predictor of MVO_2_ [33]. Despite the strong pathophysiological rationale reported above, the estimation of myocardial O_2_ with the double product might be imprecise, especially in conditions of acute hemodynamic manipulations [34, 35], but it seems more reliable in steady-state conditions [36].

In our analysis, MEEi progressively declines with increasing levels of insulin-resistance, a relation that is maintained also after multiple adjustments for potential confounders. This result was expected. We had already seen that in the treated hypertensive patients of the Campania Salute Network registry [20], metabolic syndrome and type 2 diabetes were associated with lower levels of MEEi, with the worst performance found when diabetes and metabolic syndrome coexisted. The hypothesis that insulin resistance could be the reason was near obvious and, interestingly, we could confirm this hypothesis in the non-diabetic population-based cohort of the SHS. Studies on substrate utilization performed with Positron Emission Tomography document that insulin resistance in diabetes and obesity is in fact associated with increased fatty acid utilization [37], thus substantially reducing the ratio between high energy phosphate production and number of O_2_ used [21–23], consistent with our clinical evidence of a reduced mechanical energetic efficiency with progressing degrees of insulin resistance. Interestingly this energetic mismatch is also demonstrated in type 1 diabetes [38], providing additional direct evidence for the role of insulin.

There is a large number of studies demonstrating a link between insulin resistance and non-ischemic cardiomyopathy or even heart failure [39, 40]. In another analysis in the SHS cohort, using acute myocardial infarction as a competing risk event, we found that the hazard of heart failure with type 2 diabetes was even higher than with arterial hypertension [41]. Despite the presence of many cardiovascular characteristics associated with incident heart failure, diabetes remained a potent determinant of risk of heart failure, indicating that non-hemodynamic characteristics participate to the biological profile at risk of heart failure [42]. Our study suggests that abnormality of mechanisms of production of energy related to insulin-resistance might be an important link to explain evolution toward heart failure. Interestingly, in the presence of increasing insulin resistance, increase in LV mass is more evident than increase in stroke volume. As documented in many previous studies, in the presence of normal LV systolic function at the chamber level, variations of LV mass tend to parallel variations of stroke volume [15, 43], because of a coherent increase in wall thickness and chamber volume. In our case, this parallelism is altered by the greater increase in wall thickness than in LV chamber dimension, as documented by the progressive increase in relative wall thickness, and in heart rate. From the hemodynamic standpoint, this plot makes understandable why progressive insulin-resistance parallels increased O_2_ consumption without a corresponding increased LV pump performance, therefore decreasing myocardial energetic efficiency.

Also, consistent with our findings, insulin resistance has also been reported to be associated with other adverse characteristics of CV system, including diastolic dysfunction [44] and increased arterial stiffness [45]. In addition to the impaired regulation of substrate metabolism and delivery, other mechanisms are also involved in the association of insulin resistance with incident CV disease, including alterations of signal transduction [6].

## Conclusion

The present study demonstrated that myocardial energetic efficiency is affected by the level of insulin resistance in non-diabetic participants from the SHS. Patients with high insulin-resistance have low amount of blood, ejected at each systole, per gram of LV mass, independently of common confounders such as hypertension, waist circumference and markers of inflammation.

## Additional file

**Additional file 1: Table S1.** LV geometry, systolic function and performance in quartiles of HOMA-IR.

## Abbreviations

CV: cardio vascular
LV: left ventricle
LVH: left ventricular hypertrophy
MEE: mechano-energetic efficiency
MVO_2_: myocardial oxygen consumption
SBP: systolic blood pressure
SV: stroke volume
SW: stroke work
