# Supplementary material for: Myocardial mechano-energetic efficiency and insulin resistance in non-diabetic members of the Strong Heart Study cohort
Source: Cardiovasc Diabetol. 2019 Apr 30;18:56. doi: 10.1186/s12933-019-0862-9 (PMC6492323; doi:10.1186/s12933-019-0862-9)
Supplement: Supplementary file 1 — Additional file 1: Table S1. LV geometry, systolic function and performance in quartiles of HOMA-IR. [file 12933_2019_862_MOESM1_ESM.docx]

| **Table S1:** LV geometry, systolic function and performance in quartiles of HOMA-IR | | | | |
| --- | --- | --- | --- | --- |
| Variables | ≤1.71  (n=784) | 1.72-2.75  (n=776) | 2.76-4.66  (n=785) | ≥4.67  (n=783) |
| LV mass index (g/m^2.7^) † | 35.4±8.2 | 37.2±8.5 | 39.2±8.7 | 41.9±9.8 |
| *Relative wall thickness †* | *0.300±0.048* | *0.308±0.048* | *0.315±0.047* | *0.32±0.047* |
| Ejection fraction (%) | 62.8±5.4 | 63.2±5 | 63±5.1 | 63.1±5.7 |
| Midwall shortening (%)† | 18.1±2.1 | 18.0±1.9 | 17.7±1.9 | 17.6±2.1 |
| Stroke index (ml/m^2.04^) † | 25.9±4 | 26.4±4 | 26.5±4 | 27.6±4.4 |
| Cardiac index (l/m^1.83^) † | 1.93±0.4 | 2.0±0.4 | 2.0±0.4 | 2.2±0.4 |

† p for linear trend<0.0001.
